# Physicians’ Knowledge, Altitudes, and Perceived Barriers of Inappropriate Prescribing for Older Patients in Shanghai, China

**DOI:** 10.3389/fphar.2022.821847

**Published:** 2022-08-22

**Authors:** Jing Yuan, Guizhi Yin, Meng Gu, Kevin Z. Lu, Bin Jiang, Minghui Li

**Affiliations:** ^1^ Minhang Hospital and Department of Clinical Pharmacy at School of Pharmacy, Fudan University, Shanghai, China; ^2^ Department of Cardiology, Minhang Hospital, Fudan University, Shanghai, China; ^3^ University of South Carolina College of Pharmacy, Columbia, SC, United States; ^4^ Department of Pharmacy Administration and Clinical Pharmacy, School of Pharmaceutical Sciences, Peking University, Beijing, China; ^5^ Department of Clinical Pharmacy and Translational Science, University of Tennessee Health Science Center, Memphis, TN, United States

**Keywords:** potentially inappropriate medication, inappropriate prescribing, adverse drug event, surveys and questionnaires, physicians

## Abstract

**Background:** Inappropriate medication use is common around the world, particularly among older patients, and, despite potentially being preventable, often leads to adverse clinical and economic outcomes. However, there is a dearth of information regarding this prominent issue in China.

**Objectives:** To evaluate the extent to which the physician can correctly identify potentially inappropriate medication (PIM) in older patients and to understand physicians’ attitudes towards improving PIM knowledge.

**Methods:** An online, cross-sectional survey was conducted anonymously among practicing physicians in China from November through December 2020. Knowledge of PIM was accessed using seven clinical vignettes covering a wide variety of therapeutic areas. Source of information and perceived barriers regarding PIM were also evaluated. We performed the ordinary least square regression analysis to understand the potential factors related to physicians’ knowledge of PIM.

**Results:** A total of 597 study participants were included in the analysis. More than half of them had never heard of any screening tool for PIMs (*n* = 328, 54.9%) and the most frequently acknowledged tool was the China PIM Criteria (*n* = 259, 43.4%). For the seven clinical vignettes testing physicians’ knowledge on the medications that should be generally avoided in older patients, the mean score was 2.91 points out of 7 (SD: 1.32), with the median score of three points (IQR: 2–4). Only one-third of the respondents were feeling confident when prescribing for older patients (*n* = 255, 35.08%). Package inserts have been used as the major source of PIM information (always, *n* = 177, 29.65%; frequently, *n* = 286, 47.91%). Perceived barriers to appropriate prescribing include polypharmacy (*n* = 460, 77.05%), lack of formal education on prescribing for the older patients (*n* = 428, 71.69%).

**Conclusion:** In this online survey evaluating physicians’ ability to detect PIM for older patients, approximately 40% of PIM were recognized, suggesting an insufficient level of knowledge about appropriate prescribing.

## Highlights

• Inappropriate medication use is common around the world, particularly among older patients. However, there is a dearth of information regarding this prominent issue in China.
• In this online survey evaluating physicians’ ability to detect PIM for older patients in China, more than half of PIM was unrecognized, leading to concerns over the appropriate prescribing in China.
• Even though multiple PIM screening tools were developed to assist physicians to detect PIMS, physicians’ awareness of the PIM screening tool is not ideal.
• More appropriate strategies, e.g., continuing medical education, should be explored to improve the quality of prescribing provided by doctors for older patients.

## Introduction

Potentially inappropriate medication (PIM) refers to the medication, of which the known risk outweighs its expected benefit, or there are medications with favorable risk-benefit ratios (Beers et al., 1991). Currently, inappropriate medication use is common worldwide, particularly among older patients (Fialová et al., 2005; Clark et al., 2020; Fujie et al., 2020), and, despite potentially being preventable, often causes risk of adverse health outcomes, such as hospitalizations, mortality, cognitive, and functional impairments (Cahir et al., 2010; Varga et al., 2017; Jeon et al., 2018; Sunaga et al., 2020). In China, the prevalence of irrational prescribing was more than 50%, causing approximately 200,000 death every year.

Defective choice of medications made by physicians is the major cause of inappropriate prescribing, particularly for older patients (Simonson and Feinberg, 2005). The evidence gap in geriatric care further impedes the ability of physicians to make sound prescribing decisions by forcing them to infer from data collected from younger patients. Older patients are generally under-represented in the clinical trials that investigate the efficacy and safety of drugs, leading to insufficient evidence for these special populations (Levit et al., 2018). Compared to the general adult population, older adults may have altered pharmacokinetic and pharmacodynamic characteristics, leading to increased vulnerability to serious drug-related risks, such as adverse drug reactions, drug-drug interactions, and drug-disease interactions (Davies and O'Mahony, 2015; Patel and Patel, 2018).

Over the past decades, there were several screening tools developed to assist prescribers in detecting PIM in older patients, including Beer’s Criteria developed in the United States (U. S) (Beers et al., 1991; Fick et al., 2003; American Geriatrics Society Beers Criteria Update Expert, 2012), Screening Tool of Older Persons’ potentially inappropriate Prescriptions (STOPP) and Screening Tool to Alert doctors to Right Treatment (START) developed in the United Kingdom (U. K) (O'Mahony et al., 2015; Gallagher et al., 2008), and PIM-Check developed in France and Switzerland (Desnoyer et al., 2017). These screening tools, providing an explicit list of drugs that should generally be avoided or used with caution in older patients, (Beers et al., 1991) has shown to be effective in reducing PIMs (Lavan et al., 2016). Since variations in local language and healthcare system may exist between different countries, few developing countries, such as Thailand (Almeida et al., 2019) and Brazil (Prasert et al., 2018), also developed their own screening tools for inappropriate medications in older patients. In 2017, the China Geriatric Health Care Society released the *China*’*s elderly potential inappropriate drug use judgment standard*, which is the first Chinese-version PIM screening tool. The China PIM criteria were established based on nine sets of criteria published across the world (United States, Canada, Japan, France, Norway, Germany, South Korea, Austria, Thailand, and Chinese Taiwan) and have been validated through a two-round modified Delphi process. It has been slowly applied in the prescribing practice.

Even though physician plays a key role in appropriate prescribing for older patients, few studies evaluated physicians’ ability in detecting PIM (Maio et al., 2011; Ramaswamy et al., 2011; Fadare et al., 2019). However, there is a dearth of information regarding this prominent issue in China. Physicians are required to have an MBBS (Bachelor of Medicine, Bachelor of Surgery) degree to prescribe medications in China. Based on the limited data, the PIM is highly prevalent in China. In 2019, the National Health Commission (NHC) released the *Opinions on Strengthening the Rational Use of Medicines* to enhance appropriate prescribing (Bulletin of the National Health Commission P, 2020).

### Objective

The main objective of this study was: (Beers et al., 1991) to evaluate the extent to which the physician can correctly identify PIM in older patients; (Fujie et al., 2020) to describe the source of PIM information obtained by physicians; and (Clark et al., 2020) to understand physicians’ attitudes towards improving PIM knowledge.

## Materials and Methods

### Study Design

We carried out a cross-sectional survey on an online platform managed by Wenjuanxing (https://www.wjx.cn/) through WeChat messages in China. The survey was available online to study participants from November through December 2020. We invited physicians who practiced in either community clinics or hospitals to participate. Ethical approval was obtained from the Institutional Review Boards of Minhang Hospital of Fudan University. The documentation of informed consent was waived for this study because it employed an anonymous survey approach. This study followed the Checklist for Reporting Results of Internet E-Surveys (CHERRIES) (Eysenbach, 2004).

### Survey Questionnaire

The questionnaire consisted of demographic data questions and clinical vignettes related to the knowledge and perceptions of PIM adapted from the previous survey (Maio et al., 2011; Ramaswamy et al., 2011; Fadare et al., 2019). The questionnaire was also modified to specifically reflect the delivery of healthcare and the therapeutic choices available in China. An expert panel consisting of physicians, pharmacists, and outcomes researchers was organized to review the clarity and accuracy of survey questions. Based on expert opinions, we revised some survey questions to improve quality and avoid misunderstanding.

The questionnaire included four parts; the first section was to collect the physicians’ characteristics, including gender, age, region, education, years of practice, and department. The second section was to collect the source of PIM information and physicians’ subjective assessment of their PIM knowledge. To access physicians’ confidence when prescribing for older patients, the participants were asked to rate their statement from strongly disagree (Beers et al., 1991) to strongly agree (Varga et al., 2017) *via* a 5-point Likert scale (Ramaswamy et al., 2011). The third part was to access the physicians’ knowledge level of PIM *via* seven clinical vignettes using the PIM-China as reference. Participants were expected to choose the best answer for the multiple-choice question about the medication that should be avoided in older patients. The clinical vignettes covered a wide variety of therapeutic areas, including hypertension, atrial fibrillation, depression, anxiety, insomnia, arthritis, and stroke. The clarity and content validity of the vignettes have been reviewed and approved by experts in clinical pharmacy and geriatrics medicine. The fourth part was to capture the barriers faced by the physicians to prevent PIM in older patients. Perceived barriers were assessed *via* a 5-point Likert scale.

### Participants Recruitment

As described in the previous study (Sun et al., 2017), to obtain a more representative sample of physicians, a total of twenty clinical pharmacists were chosen as the initial deliverers, who invited physicians from their units to participate in the survey. The invitation to participate was distributed *via* WeChat private messages. As the largest social media platform in China, WeChat has been widely used to publish online surveys (Hua et al., 2020; Xu et al., 2020). To avoid multiple answers from the same participant, each WeChat account was only allowed to respond to the questionnaire once. A total of 900 physicians have been identified working in the same units as the initial deliverers and were invited to participate in the survey. Among them, 738 physicians have responded to the survey. The response rate was 82.0%.

### Selection Criteria

The returned questionnaires were deemed to be eligible if all the questions were answered. The questionnaires were excluded from the analysis if the participants submitted the same answers for all the seven clinical vignettes.

### Data Analysis

For descriptive statistics, numbers and percentages were reported for categorical variables, means, and standard deviation for continuous variables. We used the Chi-square test to compare categorical variables and *t*-test for continuous variables. For the seven clinical vignettes, we calculated the mean score for each participant. We also performed an ordinary least square regression analysis to evaluate the potential factors influencing physicians’ knowledge of PIM. In the regression model, the score obtained from the clinical vignettes was used as the dependent variable, and the physicians’ characteristics and source of information were included as independent variables. Statistical significance was determined at a level of 0.05. All statistical analyses were performed using SAS 9.4 (SAS Institute Inc., Cary, NC).

## Results

### Characteristics of Respondents

After excluding 141 study participants that were deemed ineligible based on the selection criteria, 597 (or 80.9%) of 738 physicians were included in the analysis. As shown in Table 1, the majority of respondents were aged between 30 and 39 years old (*n* = 257; 43.1%). 381 (or 63.8%) respondents were female, and 230 (or 38.5%) held a graduate degree. Nearly half of the respondents worked in community hospitals (*n* = 275; 46.1%) and have practiced for 10–19 years (*n* = 221; 37.0%). 291 (or 48.7%) of the respondents practiced internal or family medicine. Other specialties that can prescribe medications in China include emergency medicine, oncology, endocrinology, psychiatry, and others.

**TABLE 1:** Self-reported characteristics of physicians who participated in the survey.

Characteristics	Number (n)	Percentage (%)
Age		
20–29	118	19.8
30–39	257	43.1
40–19	170	28.5
50+	52	8.7
Gender		
Male	216	36.2
Female	381	63.8
Education		
MBBS degree	367	61.5
Graduate degree	230	38.5
Type of hospital		
Community hospital	275	46.1
Secondary hospital	59	9.9
Tertiary hospital	220	36.9
Private hospital/others	43	7.2
Years of practice		
<5	155	26.0
5–9	102	17.1
10–19	221	37.0
20–30	86	14.4
>30	33	5.5
Specialty		
Internal/general medicine	291	48.7
Emergency medicine	51	8.5
Others	255	42.7
Percentage of patients aged over 60		
<10%	62	10.4
10–24%	45	7.5
25–49%	89	14.9
≥50%	401	67.2
Confidence in prescribing for the elderly		
Strangle agree	34	4.68
Agree	221	30.40
Neutral	383	52.68
Disagree	79	10.87
Strongly disagree	10	1.38

MBBS, bachelor of medicine, Bachelor of Surgery.

Of all physicians who responded to the survey, only one-third of the respondents agreed with the statement that they were feeling confident when prescribing for older patients (Table 1; strongly agree, *n* = 34, 4.68%; agree, *n* = 221, 30.40%), whereas 12.25% of them feeling unconfident in their prescribing practice (disagree, *n* = 79, 10.87%; strongly disagree, *n* = 10, 1.38%).

### Awareness and Knowledge of Potentially Inappropriate Medication

**FIGURE 1:**
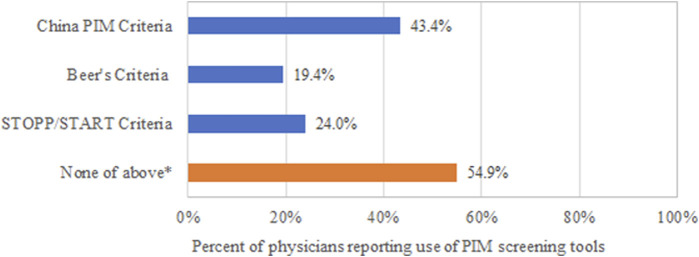
Respondents knowledge of the PIM criteria.

More than half of the respondents had never heard of the screening tool for PIMs (Figure 1; *n* = 328, 54.9%). Among the physicians who responded to the survey, the most frequently acknowledged tool was the China PIM Criteria (*n* = 259, 43.38%), followed by the STOPP/START Criteria (*n* = 143, 23.95%) and Beer’s criteria (*n* = 116, 19.43%).

**FIGURE 2:**
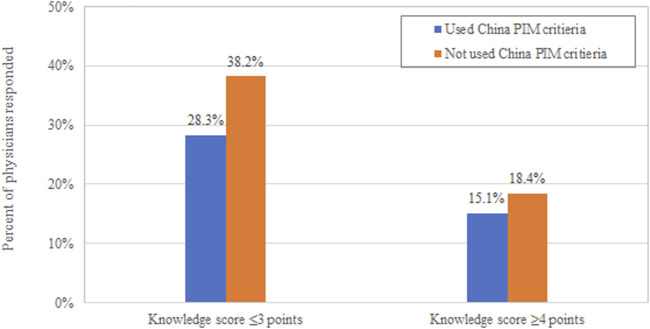
Physician’s response to clinical vignettes.

Respondents were tested in the seven clinical vignettes covering the prescribing for cardiovascular (stroke, hypertension, and atrial fibrillation), psychological disorders (anxiety, depression, and insomnia), and arthritis. The mean score was 2.91 points out of 7 (SD: 1.32); the median score was three points (IQR: 2–4). Only one-third of the respondents (*n* = 300; 33.50%) scored four or more out of seven, whereas only 10% of physicians (*n* = 69; 11.56%) answered five or more vignettes correctly. Physicians who used China PIM criteria had higher scores than those did not use such tool (Figure 2).

**FIGURE 3:**
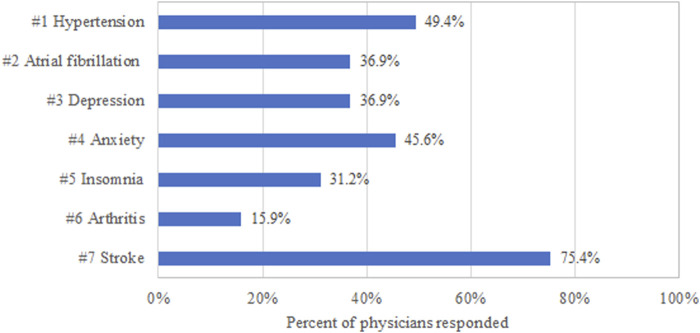
Physicians’ vignette-based scores by their knowledge of PIM criteria.

Among the seven vignettes, the respondents scored highest for the vignettes related to stroke (Figure 3; *n* = 450, 75.38%), following by arthritis (*n* = 95, 15.91%), insomnia (*n* = 186, 31.16%), anxiety (*n* = 272, 45.56%), depression (*n* = 220, 36.85%), atrial fibrillation (*n* = 220, 36.85%), and hypertension (*n* = 295, 49.41%).

### Source of Information

Table 2 shown the respondents’ source of information. Package inserts were the most commonly used information source for PIM; 77.56% of respondents agreed with the statement they always (*n* = 177, 29.65%) or frequently (*n* = 286, 47.91%) check package inserts if having questions regarding the drug products. The less commonly used information sources were knowledge base in English (*n* = 113, 18.93%), consultation with clinical pharmacists (*n* = 170, 28.48%), knowledge base in Chinese (*n* = 241, 40.37%), medical textbooks (*n* = 275, 46.06%), and Internet or mobile Apps (*n* = 303, 50.75%).

**TABLE 2:** Source of PIM information.

	Always	Frequent	Sometimes	Seldom	Never
Knowledge base in Chinese	65 (10.89%)	176 (29.48%)	200 (33.50%)	125 (20.94%)	31 (5.19%)
Knowledge base in English	29 (4.86%)	84 (14.07%)	165 (27.64%)	134 (22.45%)	185 (30.99%)
Package inserts	177 (29.65%)	286 (47.91%)	113 (18.93%)	19 (3.18%)	2 (0.34%)
Internet or mobile Apps	76 (12.73%)	227 (38.02%)	206 (34.51%)	71 (11.89%)	17 (2.85%)
Clinical pharmacists	37 (6.20%)	133 (22.28%)	247 (41.37%)	135 (22.61%)	45 (7.54%)
Medical textbooks	67 (11.22%)	208 (34.84%)	214 (35.85%)	94 (15.75%)	14 (2.35%)

### Barriers to Appropriate Prescribing

When asked about the barriers impeding appropriate prescribing in older patients, the top barriers agreed by respondents were polypharmacy (77.05%), lack of formal education on prescribing for the elderly (71.69%), lack of information about which medications a patient is already taking (65.33%), no access to medical records (59.30%) and limited time (57.45%) (Table 3).

**TABLE 3:** Identified barriers against appropriate prescribing for the elderly.

	Strongly agree	Agree	Neutral	Disagree	Strongly disagree
Limited time	64 (10.72%)	279 (46.73%)	212 (35.51%)	40 (6.70%)	2 (0.34%)
Lack of communication	48 (8.04%)	200 (33.50%)	268 (44.89%)	75 (12.56%)	6 (1.01%)
No access to medical records	66 (11.06%)	288 (48.24%)	206 (34.51%)	37 (6.20%)	0 (0.00%)
Unwillingness to discontinue a medication	69 (11.56%)	269 (45.06%)	228 (38.19%)	28 (4.69%)	3 (0.50%)
Patient request to begin a specific medication	50 (8.38%)	236 (39.53%)	257 (43.05%)	50 (8.38%)	4 (0.67%)
Lack of formal education on prescribing for the elderly	115 (19.26%)	313 (52.43%)	147 (24.62%)	22 (3.69%)	0 (0.00%)
Cost of medication to patient	37 (6.20%)	180 (30.15%)	280 (46.90%)	93 (15.58%)	7 (1.17%)
Not covered by insurance	39 (6.53%)	176 (29.48%)	261 (43.72%)	114 (19.10%)	7 (1.17%)
Lack of information about which medications a patient is already taking	76 (12.73%)	314 (52.60%)	185 (30.99%)	21 (3.52%)	1 (0.17%)
Lack of acceptable therapeutic alternatives	44 (7.37%)	229 (38.36%)	255 (42.71%)	66 (11.06%)	3 (0.50%)
Lack of clinical pharmacist	93 (15.58%)	232 (38.86%)	207 (34.67%)	63 (10.55%)	2 (0.34%)
Polypharmacy	148 (24.79%)	312 (52.26%)	116 (19.43%)	20 (3.35%)	1 (0.17%)

### Factors Associated With Knowledge of Potentially Inappropriate Medication

In the regression analysis, the statistically significant factors influencing physicians’ knowledge of PIM included working in private hospital/others (Table 4; *p* = 0.039) and having 50% or more of their patients that were aged over 60 (*p* = 0.003).

**TABLE 4:** Factors associated with the knowledge level of potentially inappropriate medication.

Characteristics	Estimate	Standard error	*p*-value
Age			
20–29	Ref	-	-
30–39	0.12	0.19	0.540
40–19	0.05	0.25	0.858
50+	0.16	0.38	0.678
Gender			
Male	Ref	-	-
Female	−0.13	0.12	0.255
Education			
MBBS degree	Ref	-	-
Graduate degree	0.20	0.13	0.134
Years of practice			
<5	Ref	-	-
5–9	−0.09	0.19	0.639
10–19	−0.20	0.20	0.330
20–30	−0.01	0.28	0.974
>30	−0.35	0.42	0.403
Type of hospital			
Community hospital	Ref	-	-
Secondary hospital	0.15	0.19	0.455
Tertiary hospital	0.01	0.15	0.966
Private hospital/others	0.47	0.23	0.039
Specialty			
Internal/general medicine	Ref	-	-
Emergency medicine	−0.14	0.21	0.507
Others	−0.14	0.13	0.277
Percentage of patients aged over 60			
<10%	Ref	-	-
10–24%	−0.12	0.26	0.654
25–49%	0.24	0.23	0.291
≥50%	0.60	0.20	0.003
Used China PIM criteria			
No	Ref	-	-
Yes	0.16	0.11	0.151

MBBS, bachelor of medicine, Bachelor of Surgery; PIM, potentially inappropriate medication.

## Discussion

To our best knowledge, this is the first study evaluating physicians’ knowledge of PIM in China. In this large, online survey, respondents seemed to possess an inadequate knowledge for geriatric prescribing, even though they prescribed for a large proportion of older patients in their practice. The knowledge gap in the PIM has been reinforced in earlier studies, despite the intrinsic limitations associated with the vignette-based survey (Nightingale et al., 2015). Recent studies indicated that higher scores were achieved by doctors in the knowledge evaluation *via* the clinical vignettes compared to medical record review (Peabody et al., 2004; Veloski et al., 2005). By using seven vignettes testing doctors’ prescribing ability, we found that only one out of three physicians achieved an acceptable score (4 out of 7). More importantly, less than half of them has heard about the China PIM criteria, which has been released for more than five years, suggesting that the awareness of these useful PIM screening tool is not sufficient among the practicing physicians.

Our results are similar to previous studies on PIM in China. Approximately half of older patients admitted to hospitals have experienced at least one PIM (Mo et al., 2016; Zhang et al., 2021). In a survey of Chinese community doctors, it was found that prescribing PIMs to older patients was very common. The probability of exposure to at least one high-risk PIM was 6 per 1,000, and the low-risk PIM exposure rate is 117.5 per 1,000 (Fu et al., 2020). Hence, there is an urgent need to improve prescribing quality for older patients.

Despite the overall unsatisfactory performance in detecting PIM, the average scores for the clinical vignettes were varied largely by therapeutic classes. In this analysis, only 15.9% of respondents were able to provide correct answers for older patients with arthritis, while they scored 75.4% for stroke-related scenarios. It may be explained by the fact that stroke is a more serious disease than arthritis, and hence physicians may pay more attention or receive more training in cardiovascular disease. Physicians’ performance in detecting PIM is also corrected to their awareness of the PIM screening tool, even though it does not reach statistical significance. Another concerning finding produced by this survey is that more than half of physicians obtained their PIM information from package inserts. Even though the package inserts were approved by NMPA, important drug information, such as drug-drug interaction and potential adverse reactions for older patients, may be outdated. Still, more than 10% of physicians browsed internet or mobile Apps, which is not ideal in quality of information.

Considering the serious harm of PIM, it is imperative to improve physicians’ knowledge of appropriate prescribing in older patients especially in countries like China, which has entered an accelerated period of the aging population. It was projected that by 2050, the number of older people in China will reach 400 million (Fang et al., 2015). With the rapid aging of the population leading to an increasing proportion of patients with multiple comorbidity and polypharmacy, improving physicians’ awareness and knowledge of geriatric should receive more attention in China. Therefore, it is warranted to develop more effective intervention to improve appropriate prescribing in China.

### Future Implications

The key findings from our survey could inform policymakers to better design healthcare systems, contributing to the improved quality of care in China.

First, to address the issue of low level of appropriate prescribing, multi-dimensional strategies should be implemented. For example, geriatric pharmacology should be incorporated into continuing medical education to improve physicians’ knowledge of geriatric prescribing. A multifaceted educational program should be developed to raise physicians’ awareness of the availability of PIM criteria. An incentive program should be designed to encourage the use of PIM criteria in prescribing practices for older patients.

Second, assessment of the barriers impeding physicians from appropriate prescribing may contribute to the appropriateness of prescribing by identifying the long-standing systemic pain point that requires further improvement. The lack of clinical pharmacists has been recognized as the biggest barrier to achieving appropriate prescribing by the majority of the respondents. Across the continuum of pharmaceutical care, pharmacists should play a central role in ensuring drug safety, while their role has been mainly focused on drug dispensing in China. The NHC published the *Opinion on Strengthening Pharmacy Administration and Transferring Pharmaceutical Care* in 2017 (Chinese Society of Geriatric Health Care CGS et al., 2018). The *Opinion* suggested expanding the pharmacist’s role from prescription dispensing to prescription review. This survey revealed that more than half of physicians rely on uncensored information for their prescription, further reinforcing the urgency to enhance the pharmacist’s role in the prescription review.

Third, since the HIS system has been implemented in most secondary and tertiary hospitals in China, with some hospitals piloting the clinical decision support system, it would be feasible to implant the PIM screening tools into such a system to alert prescribers.

### Limitations

Several limitations should be considered. First, we developed clinical vignettes with a primary focus on the Beer’s and China PIM criteria. Even though we included an expert panel to adapt the survey questionnaire, the instrument is not validated. These clinical vignettes only represent a small proportion of prescription scenarios, and hence, physicians’ knowledge level of PIM may be inadequately reflected in this analysis. Second, our findings may not be generalizable to other practicing physicians in China. Despite our efforts made to recruit a large number of physicians, the respondents only accounted for a small proportion of doctors in China. In addition, the study sample might not be representative to the physicians practicing in Shanghai, China, with a larger proportion of young practitioners, females, and having graduate degrees. The knowledge of inappropriate prescribing in other groups might be different. Finally, we cannot exclude the possibility of selection bias because physicians who were confident with PIM were more likely to participate in the survey. As such, it would be possible to include patients with better performance in detecting PIM.

## Conclusion

In summary, this study is the first, to our knowledge, to investigate physicians’s knowledge level of appropriate prescribing for older adults in China. Our findings indicate an unsatisfactory knowledge level about appropriate prescribing for older patients among Chinese physicians. Considering the potential harms of PIM, more effective strategies to improve awareness and knowledge about PIM are urgently needed.

## Data Availability

The raw data supporting the conclusion of this article will be made available by the authors, without undue reservation.
